# Radiomic Texture and Shape Descriptors of the Rectal Environment on Post-Chemoradiation T2-Weighted MRI are Associated with Pathologic Tumor Stage Regression in Rectal Cancers: A Retrospective, Multi-Institution Study

**DOI:** 10.3390/cancers12082027

**Published:** 2020-07-24

**Authors:** Charlems Alvarez-Jimenez, Jacob T. Antunes, Nitya Talasila, Kaustav Bera, Justin T. Brady, Jayakrishna Gollamudi, Eric Marderstein, Matthew F. Kalady, Andrei Purysko, Joseph E. Willis, Sharon Stein, Kenneth Friedman, Rajmohan Paspulati, Conor P. Delaney, Eduardo Romero, Anant Madabhushi, Satish E. Viswanath

**Affiliations:** 1 Department of Biomedical Engineering, Case Western Reserve University, Cleveland, OH 44106, USA; calvarezj@unal.edu.co (C.A.-J.); jta35@case.edu (J.T.A.); kxb413@case.edu (K.B.); kenneth.friedman@case.edu (K.F.); anant.madabhushi@case.edu (A.M.); 2 Computer Imaging and Medical Application Laboratory, Universidad Nacional de Colombia, Bogotá 111321, Colombia; edromero@unal.edu.co; 3 Department of Biomedical Engineering, Georgia Institute of Technology, Atlanta, GA 30332, USA; ntalasila@gatech.edu; 4 Department of General Surgery, University Hospitals Cleveland Medical Center, Cleveland, OH 44106, USA; justin.brady@uhhospitals.org (J.T.B.); sharon.stein@uhhospitals.org (S.S.); 5 Department of Abdominal Imaging, University Hospitals Cleveland Medical Center, Cleveland, OH 44106, USA; jaykrishna.gollamudi@uhhospitals.org; 6 Louis Stokes Cleveland Veterans Affairs Medical Center, Cleveland, OH 44106, USA; eric.marderstein@va.gov; 7 Department of Colorectal Surgery, Cleveland Clinic, Cleveland, OH 44106, USA; KALADYM@ccf.org (M.F.K.); delanec@ccf.org (C.P.D.); 8 Section of Abdominal Imaging and Nuclear Radiology Department, Cleveland Clinic, Cleveland, OH 44195, USA; puryska@ccf.org; 9 Department of Pathology, University Hospitals Cleveland Medical Center, Cleveland, OH 44106, USA; JosephE.Willis@uhhospitals.org; 10 Department of Radiology, University Hospitals Cleveland Medical Center, Cleveland, OH 44106, USA; RajMohan.Paspulati@uhhospitals.org

**Keywords:** radiomics, rectal cancer, texture, shape, magnetic resonance imaging, treatment response, machine learning

## Abstract

(1) *Background*: The relatively poor expert restaging accuracy of MRI in rectal cancer after neoadjuvant chemoradiation may be due to the difficulties in visual assessment of residual tumor on post-treatment MRI. In order to capture underlying tissue alterations and morphologic changes in rectal structures occurring due to the treatment, we hypothesized that radiomics texture and shape descriptors of the rectal environment (e.g., wall, lumen) on post-chemoradiation T2-weighted (T2w) MRI may be associated with tumor regression after neoadjuvant chemoradiation therapy (nCRT). (2) *Methods*: A total of 94 rectal cancer patients were retrospectively identified from three collaborating institutions, for whom a 1.5 or 3T T2w MRI was available after nCRT and prior to surgical resection. The rectal wall and the lumen were annotated by an expert radiologist on all MRIs, based on which 191 texture descriptors and 198 shape descriptors were extracted for each patient. (3) *Results*: Top-ranked features associated with pathologic tumor-stage regression were identified via cross-validation on a discovery set (*n* = 52, 1 institution) and evaluated via discriminant analysis in hold-out validation (*n* = 42, 2 institutions). The best performing features for distinguishing low (ypT0-2) and high (ypT3–4) pathologic tumor stages after nCRT comprised directional gradient texture expression and morphologic shape differences in the entire rectal wall and lumen. Not only were these radiomic features found to be resilient to variations in magnetic field strength and expert segmentations, a quadratic discriminant model combining them yielded consistent performance across multiple institutions (hold-out AUC of 0.73). (4) *Conclusions*: Radiomic texture and shape descriptors of the rectal wall from post-treatment T2w MRIs may be associated with low and high pathologic tumor stage after neoadjuvant chemoradiation therapy and generalized across variations between scanners and institutions.

## 1. Introduction

Colorectal cancer is the third most common cancer worldwide (incidence rate of 10.2%), of which over 700,000 patients will be annually diagnosed with tumors localized to the rectum [1]. Those with locally advanced rectal cancer typically receive neoadjuvant chemoradiation therapy (nCRT) followed by total mesorectal excision (TME) surgery, as the standard-of-care treatment protocol in the US [2]. While the goal of nCRT is to down-stage rectal tumors prior to surgery (occurring in ~50*–*60% of rectal tumors [3]), determining the exact extent of tumor regression after nCRT is critical for better personalizing interventions in rectal cancers. For instance, minimally invasive procedures [4] could be adopted in patients exhibiting marked tumor stage regression (T-stage 0*–*2, with minimal or no tumor extent within the rectal wall), thus reducing associated morbidities of bleeding or infections [5,6]. By contrast, chemoradiated tumors which still extend outside of the rectum into the perirectal fat and surrounding structures (T-stage 3*–*4) need to be accurately targeted for aggressive resection [7] and could be recommended adjuvant therapy to ensure optimal patient survival [8].

Magnetic resonance imaging (MRI) is routinely acquired both prior to as well as following nCRT to non-invasively assess rectal tumor extent in vivo [9]. As compared to less routinely used dynamic [10] or diffusion [11] MRI sequences which capture functional information, the clinically standard T2-weighted (T2w) MRI sequence offers high-resolution in vivo structural detail of the rectum and surrounding structures (lumen, mesorectum). Under the current protocol where all patients undergo surgery, post-nCRT T2w MRI is used to identify disease extent, plan surgical procedures, and thus guide patient management [9]. However, expert restaging of tumor extent on post-nCRT T2w MRI has relatively poor agreement with “ground truth” pathologic stage (MRI sensitivity of ~52% when compared to excised specimens) due to the difficulties in visual identification of residual tumor on imaging [12,13]. This confounded appearance is primarily due to the appearance of fibrotic regions within the rectal wall after neoadjuvant therapy, which have an overlapping intensity appearance with residual tumor on T2w MRI [14,15]. This suggests a critical need for more accurate imaging markers towards enabling non-invasive evaluation of rectal cancer stage after chemoradiation (and prior to surgery).

Recent advances in the field of radiomics have demonstrated great promise for computer-extracted quantitative features from radiographic images in enabling improved disease characterization compared to using visual inspection alone [16]. Radiomics descriptors have been used in conjunction with routinely available imaging to result in accurate treatment response evaluation across different cancers; including brain [17], liver [18], head-and-neck [19], prostate [20], and lung [21]. While radiomic features of rectal tumors on baseline T2w MRIs have been evaluated for associations with pathologic response to nCRT [22], there has been relatively little work examining post-nCRT T2w MRIs alone. 

The most popular suite of radiomic features involve extracting texture responses, which could help quantify the imaging appearance of deep stromal alterations caused by fibrosis [23] appearing within the rectal wall after nCRT. Other hallmarks of nCRT impact in the rectum include changes in rectal wall thickness due to the development of tumor necrosis or inflammation [24]. Chemoradiation is also intended to reduce rectal tumor extent that had originally spread outward from the rectal wall or inward into the lumen [25], implying it could cause changes in the morphology of these structures within the rectal environment. We, therefore, hypothesized that by combining radiomic descriptors that quantify (i) textural appearance changes within the rectal wall (characterizing appearance of treatment effects) as well as (ii) morphologic shape changes of rectal structures (to capture distensions of wall or lumen) on T2w MRI, we may be able to more accurately characterize rectal tumor stage regression after nCRT. The goal of this work was to automatically differentiate between low and high pathologic stages of rectal tumors after nCRT using radiomic texture and shape radiomic descriptors derived from the post-nCRT rectal environment. These descriptors were further evaluated for their resiliency across magnetic field strengths as well as for their discriminability across multiple institutions.

## 2. Results

### 2.1. Data Description

A total of 94 patients were included in this study from across three collaborating institutions, all of whom had been treated for a clinically staged T2–T4 rectal carcinoma between August 2007 and January 2019 with standard-of-care neoadjuvant chemoradiation. Mean age was 62 years (range 30–85 years), with 61 male and 33 female patients. The discovery cohort comprised 52 studies from Inst. 1 (University Hospitals Cleveland Medical Center, UHCMC). The hold-out validation cohort comprised 42 studies: (i) 31 patients from Inst. 2 (Cleveland Clinic Foundation, CCF) and (ii) 11 patients from Inst. 3 (Louis Stokes Veterans Affairs Medical Center, VAMC). All included patients had an MRI acquired after nCRT using a T2-weighted sequence at each institution, with the scanner and imaging parameters used at each site summarized in Table 1. Rectal gel had been used to routinely prep all patients at Inst. 1 and Inst. 2 but not Inst. 3. Across the three institutions, three different scanner manufacturers and 10 different models were used for MR imaging; though the range of acquisition parameters was relatively consistent within each institution. Imaging data were acquired as a series of DICOM images saved directly from the scanners. 

### 2.2. Identifying T2w Radiomic Texture Features Associated with Pathologic Tumor Down-Staging after Chemoradiation

**Figure 1:**
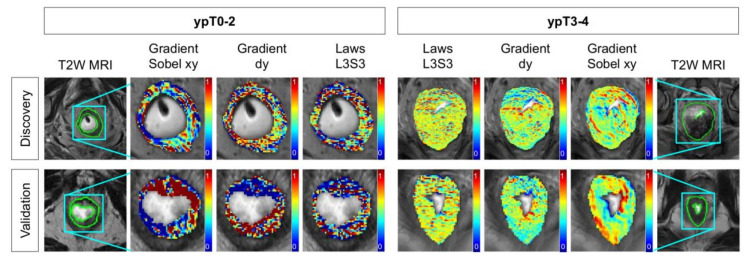
Representative radiomic heatmaps overlaid on post-treatment T2w MRI depicting texture heterogeneity differences between ypT0–2 (left) ypT3–4 (right) rectal cancer patients after long-course chemoradiation therapy. Across both discovery (top row) and validation (bottom row) cohorts, gradient and Laws responses under-express in ypT0–2 patients (more bluish-green regions) compared to ypT3–4 patients.

**Figure 2:**
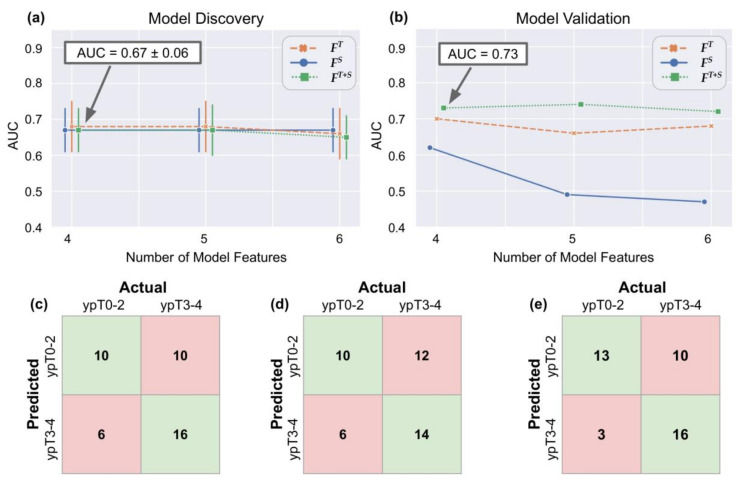
Quadratic discriminant analysis (QDA) model AUC performance while varying the number of radiomic features used (*x*-axis) when evaluated on (**a**) discovery, and (**b**) validation cohorts. The different colors and symbols correspond to ***F****^T^* (orange),***F**^S^* (blue), and ***F****^T+S^* (green); respectively. Error bars on (**a**) reflect ± 1 standard deviation of AUC in cross-validation on the discovery cohort. Also shown are confusion matrices for (**c**) ***F****^T^,* (**d**)***F**^S^*, and (**e**)***F**^T+S^* for the validation cohort at the optimized threshold. ***F****^T+S^* can be seen to result in the best overall classifier performance in terms of accurately generalizing to the validation cohort, with the optimal discrimination between pathologic stage groups achieved using 4 features.

Table 2 lists the top-ranked T2w radiomic texture features that comprise ***F****^T^* (as identified over multiple runs of 3 fold cross-validation), together with their *p*-values from Wilcoxon ranksum testing between pathologic stage groups. These features include responses to gradient and edge operators, as well as three co-occurrence statistics. Representative heatmaps for the threee top-ranked T2w texture features in Figure 1 reveal that these edge or gradient responses under-express in ypT0–2 patients (bluish-green appearance, left half) compared to significant over-expression in ypT3–4 patients (greenish-yellow appearance, right half). Based on the QDA model trends for ***F****^T^* (orange lines in Figure 2a,b while varying the number of features, using four radiomic texture features yielded an optimal discovery AUC of 0.68 ± 0.07 and hold-out validation AUC of 0.70. At the optimized threshold, this corresponded to an accuracy of 69% (MCC of 0.38) in identifying ypT0–2 patients after nCRT on the discovery cohort and 62% accuracy on the external validation cohort (63% sensitivity, 62% specificity, 0.23 MCC, Figure 2c).

### 2.3. Identifying T2w Radiomic Shape Features Associated with Pathologic Tumor Down-Staging after Chemoradiation

**Figure 3:**
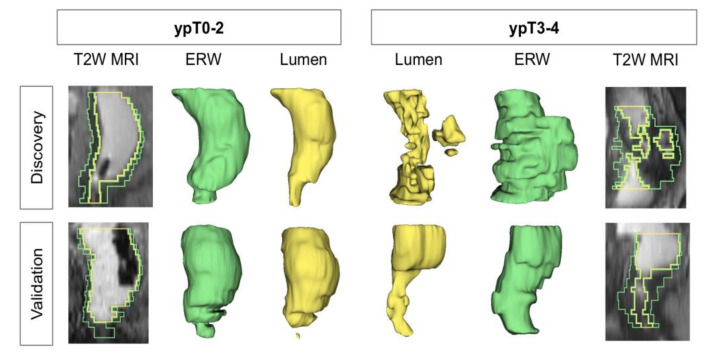
2D and 3D renderings of entire rectal wall (green) and the lumen (yellow) in the sagittal plane on T2w MR images revealing morphologic differences between post-chemoradiation ypT0–2 (left) and ypT3–4 (right) patients; across both discovery (top row) and validation (bottom row) cohorts. Higher pathologic tumor stages are characterized by thicker rectal walls and less continuous lumen structures.

The top-ranked T2w radiomic shape features in ***F****^S^* comprise an equal number of rectal wall and lumen features (Table 2). Two-dimensional and 3D renderings of the entire rectal wall (green) and lumen (yellow) in Figure 3 reveal that ypT3–4 tumors are associated with thicker rectal walls which vary in thickness across the volume; as quantified via compactness and convexity measures. ypT3–4 tumors also exhibit less continuous lumen structures with more abrupt changes across smaller volumes; as quantified by eccentricity and axis length measurements. While a QDA model trained on ***F****^S^* yielded a consistent performance on the discovery cohort (Figure 2a) AUC of 0.67 ± 0.06), this did not generalize as well as ***F****^T^* in hold-out validation (Figure 2b, AUC of 0.62). When using fouur features and at the optimized threshold, ***F****^S^* resulted in an accuracy of 67% (MCC of 0.34) in the discovery cohort and 57% accuracy on the external validation set (63% sensitivity, 54% specificity, 0.16 MCC, Figure 2d).

### 2.4. Combining T2w Radiomic Texture and Shape Features Consistently Discriminates Pathologic Tumor Stage Groupings after Chemoradiation across Institutions and Magnetic Field Strengths

A combination of four textural and two shape descriptors were identified as comprising ***F****^T+S^* (listed in Table 2), all of which were among the top-ranked features within ***F****^T^*and***F**^S^* individually. The resulting QDA model was consistent with the other feature vectors in discovery (AUC of 0.67 ± 0.06, Figure 2a) and yielded the best overall performance among the three feature vectors in hold-out validation (AUC of 0.73, Figure 2b). ***F****^T+S^* also yielded consistent performance when varying the number of radiomic features used, where using 4 features at the optimized threshold yielded an accuracy of 69% in the discovery cohort as well as 69% accuracy (MCC of 0.36) in hold-out validation (81% sensitivity, 62% specificity, 0.42 MCC); for identifying ypT0–2 patients as depicted in Figure 2e. Model trends for a random forests classifier [26] are illustrated in Figure S1, revealing similar trends in the performance of ***F****^T+S^*, ***F****^T^*, and ***F****^S^*, across discovery and validation cohorts. While the random forests model yields a slightly higher performance in the discovery cohort (AUC of 0.73 ± 0.05), it does not generalize as well as the QDA model in hold-out validation (AUC of 0.64).

Table S1 summarizes the performance of ***F****^T+S^* when using a QDA model for discriminating pathologic tumor stage groupings after chemoradiation, between sex-specific subgroups. While there are no significant differences in model performance between sexes in the discovery cohort, AUC and MCC values are markedly lower for females versus males in the validation cohort (though there were also fewer females in the validation cohort).

These results can be further interrogated via the boxplots in Figure S2a–f for each of the top-ranked radiomics descriptors within ***F****^T+S^*, depicting their trends across each of the 3 institutions. While both inst. 1 and 2 exhibited a similar trend in a majority of the descriptors, cases from Inst. 3 exhibited a differing trend for 2/6 descriptors. Correspondingly, the institution-specific confusion matrices for ***F****^T+S^* in Figure S2g,h reveal that all four of the ypT3–4 tumors from Inst. 3 were misclassified by the QDA model while only 6/22 ypT3–4 tumors were misclassified in Inst. 2.

**Figure 4:**
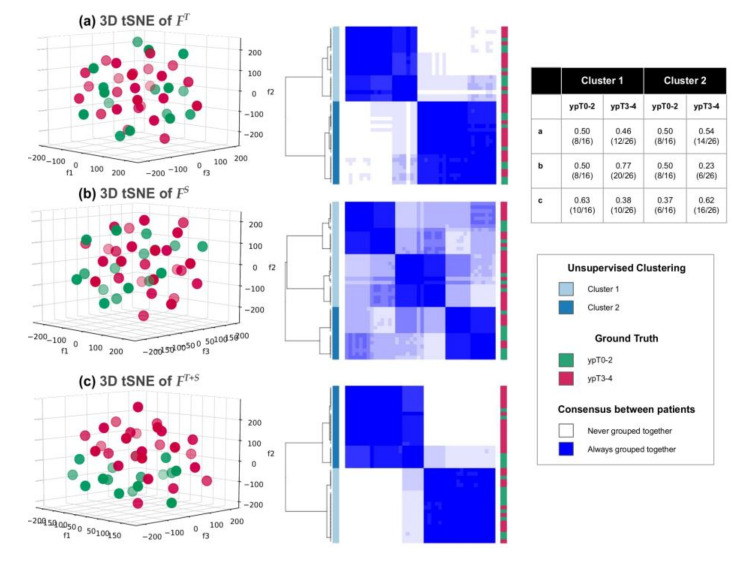
Scatter plots of t-SNE projection and consensus clustering heatmaps via (**a**) ***F****^T^*, (**b**) ***F****^S^*, (**c**) ***F****^T+S^*; in the validation cohort. Left column: 3D scatter plots of ypT0–2 tumors (green) vs. ypT3–4 tumors (red) obtained via *t*-SNE. Middle column: corresponding consensus clustering heatmaps of *t*-SNE projections (blue shading indicates the frequency with which each pair of patients was clustered together) with original ypT groupings depicted via red-green colorbar alongside. Right column: Unsupervised clustering accuracy for each *t*-SNE projection showing that ***F****^T+S^* most accurately clusters ypT0–2 from ypT3–4 tumors.

Comparing the 3D scatter plots and clustering heatmaps of t-SNE projections corresponding to each of ***F****^T^*, ***F****^S^*, and ***F****^T+S^* (Figure 4, for the validation cohort), illustrates how the combination of texture and shape descriptors most distinctively segregates pathologic T-stage groupings with 63% unsupervised clustering accuracy for both ypT0–2 and ypT3–4 tumors. By comparison, ***F****^T^* shows much weaker consensus (more varied shading in the consensus cluster heatmap) and a markedly less accurate clustering accuracy (ypT stages equally distributed across both clusters). The worst overall performance corresponds to using ***F****^S^* alone where no consistent clusters are identified, and cluster 1 comprises a majority of the cohort (50% ypT0–2 and 77% ypT3–4).

Table 3 summarizes the results of Wilcoxon ranksum testing each of the radiomic descriptors from Table 2, between 1.5 T and 3.0 T scans. No significant differences (all *p* > 0.004, Bonferroni-corrected threshold) are observed in any of the top-ranked texture and shape radiomic features between magnetic field strengths. Table 4 similarly shows the results of statistically comparing each of the top-ranked radiomics descriptors from Table 2 between 2 sets of expert annotations. No significant differences can be observed for either texture or shape descriptors derived from either wall or lumen (all *p* > 0.05) though three descriptors resulted in ICC < 0.5 (2 of which show institutional differences in Figure S2). The excellent overlap between the 2 sets of expert annotations is also reflected in relatively high DSC values, both for *R^E^* (0.72 ± 0.08) as well as *R^L^* (0.86 ± 0.1).

## 3. Discussion

In this study, we investigated the ability of radiomic features from post-treatment T2w MRI to evaluate pathologic tumor down-staging after nCRT in rectal cancers. A combination of textural and morphologic radiomic descriptors was found to most accurately distinguish between patients with ypT0–2 and ypT3–4 pathologic stages after chemoradiation, with consistent performance across discovery and hold-out validation cohorts accrued from 3 different institutions. Optimal performance of our radiomics model was achieved using 4 features, both in terms of AUC as well as accuracy of identifying ypT0–2 patients at the optimized threshold.

The most relevant T2w radiomic texture features that best differentiated low and high pathologic T-stages comprised responses to gradient and edge operators, both of which measure heterogeneity patterns in local signal intensity along the lateral axis within the rectal wall. Down-staged tumors (ypT0–2) were characterized by diminished gradient expression within the rectal wall as well as lower level-spottiness energy. Studies of the histopathology of down-staged rectal tumors after chemoradiation [27] have indicated that tumor cells are replaced by fibrosis or scar tissue, where the latter are visualized as hypo-intense regions within the rectal wall on T2w MRI [9]. We suggest that fibrosis-associated T2w signal hypo-intensities within the rectal wall of patients with tumor down-staging may be driving the subtly weakened edge and image gradients being quantified by the radiomic descriptors identified in this study. 

While shape descriptors have been previously explored in lung [28,29] and breast [30] cancers, our study is the first to evaluate this class of radiomic features for rectal structures (wall, lumen). Morphologic descriptors quantifying the thickness of rectal wall as well as the regularity of the lumen structure were found to best segregate low and high pathologic T-stages in this study. We found that the rectal wall in patients with pathologic stages T0–2 after chemoradiation was more consistently thinner (i.e., lower compactness and convexity), which intuitively aligns with definitions in the TNM system [31] in that treated tumor does not invade beyond the rectal wall, therefore minimizing distension in comparison to ypT3–4 tumors. The discontinuities and abruptness of shape variations of the lumen that were found to be associated with ypT3–4 tumors (quantified as higher eccentricity and changes in axis lengths across the rectum) likely arise based on whether disease extent after nCRT continues to intrude into the lumen [25]; potentially indicative of larger, more ulcerated tumors which did not respond to chemoradiation. Consequently, radiomic shape descriptors appear to accurately capture morphologic characteristics of tumor stage after chemoradiation in rectal cancers.

A combination of texture and shape features were identified as consistently segregating pathologic tumor stage groups, and yielded a marked improvement over reported expert restaging accuracies from the literature [12,13]. This combined model leveraged complementary information from the two types of descriptors used in this study, as evidenced when comparing scatter plots and confusion matrices resulting from using texture or shape descriptors individually. The major source of classification errors in hold-out validation stemmed from misclassification of ypT3–4 tumors accrued from one of the institutions, where these datasets were found to exhibit markedly different trends in several radiomic descriptors compared to the other institutions. In addition to using a slightly different imaging sequence (FSE vs. TSE), this institution also did not use rectal gel when preparing the patients for MR imaging. These imaging differences likely reduced the contrast between rectal wall and lumen, which may have impacted some of texture and shape radiomic descriptors which exhibited marginal differences between institutions as well as expert annotations. However, the final radiomics model largely maintained its performance across the 3 different institutions, in addition to which no significant differences were found in a majority of top-ranked radiomic descriptors when compared between different magnetic field strengths or between annotations from two different experts; suggesting the radiomics features identified in this study may be relatively resilient to annotation-based, institutional, as well as scanner differences. 

Prior related radiomics approaches for assessing treatment response in rectal cancer have primarily focused on high order texture features on pre-chemoradiation MRIs alone [22,32,33] or on quantifying texture changes between pre-, mid-, and post-treatment MRIs [34,35,36,37,38,39]. To the best of our knowledge, only one other work has examined radiomic features from post-chemoradiation T2w MRI alone [40] for evaluating pathologic complete response to therapy (ypT0N0M0). While the latter study used a comparably sized patient cohort, it utilized texture features and reported cross-validated performance on a single institution alone. Our analysis of a significantly expanded feature set also identified several co-occurrence-based features as relevant for pathologic rectal tumor response to nCRT, resonating with findings from this previous study. We have further evaluated how to combine textural and morphological radiomic descriptors of post-treatment rectal tumors to better characterize pathologic response, in a multi-institution setting. 

We do acknowledge some limitations of our study. While our final cohort was limited to slightly under 100 patients, we nevertheless performed hold-out validation on patients curated from different institutions from that of discovery. The data in this work also involved T2w sequences with different resolutions, sequences, and different scanner equipment. As the radiomics descriptors largely maintained their performance in hold-out validation despite these variations, this suggests they may be relatively robust markers of pathologic stage after chemoradiation. While sequences such as diffusion MRI have demonstrated great promise for capturing rectal tumor response [41] and T-stage [38] prior to treatment, this sequence was not consistently available for patients in our multi-institution cohort and was thus not included in our analysis. We also did not specifically assess interobserver variability in annotating the region of interest used for radiomic analysis. However, this concern may be ameliorated as we opted to characterize the entire rectal wall in-plane with the primary treated tumor location on T2w MRI, and identification of the rectal wall is far more straightforward on T2w MRI [42]. Using the rectal wall also overcomes a significant limitation of related work [34,35,36,37,40,43], all of which have utilized radiologist annotations of suspicious tumor regions on post-chemoradiation T2w MRI. The latter can be dubious [44] when there is no disease present pathologically as well as potentially suffering high interobserver variability (~50–60%) [45]. Finally, we opted to restrict our analysis to TNM staging as the criteria for pathologic outcomes after chemoradiation as tumor regression grade information was unavailable for a majority of the patients in our cohort. Despite these limitations, ours is the first multi-institution study for evaluating textural and morphological radiomic descriptors from post-treatment T2w MRIs for identifying pathologic stage groupings of rectal tumors after chemoradiation. This is a key step towards better pre-operative evaluation of rectal cancer patients in order to effectively and accurately triage them towards minimally invasive or aggressive resection procedures after chemoradiation, and thus improve their overall survival and quality of life.

## 4. Materials and Methods

### 4.1. Ethical Statement

This HIPAA-compliant, retrospective study was approved by institutional review boards (IRBs) at three institutions, University Hospitals Cleveland Medical Center (UHCMC, #07-16-40, STUDY20190073), Cleveland Clinic Foundation (CCF, #18-427), and the Louis Stokes Veterans Affairs Medical Center (VAMC, #18025-H11); with a waiver for requirement of informed consent as de-identified patient data was utilized.

### 4.2. Patient Selection

A total of 119 patients diagnosed with rectal cancer between September 2009 and October 2015 were curated from a colorectal surgery database at UHCMC. Of these, 59 patients had post-nCRT T2w MRIs available for biopsy-proven rectal adenocarcinomas, as well as having pathology reports available from examination of total mesorectal excision specimens. 6 patients were further excluded due to missing relevant information from their pathology report, and 1 patient was excluded due to poor image quality. In total, 52 patients met our initial inclusion-exclusion criteria for this study from UHCMC. A total of 137 patients diagnosed and treated for rectal cancer between August 2007 and September 2012 were curated from a colorectal surgery database at CCF. Of these, 31 patients met our inclusion-exclusion criteria of post-nCRT T2w MRIs being available after routine neoadjuvant chemoradiation and prior to total mesorectal excision, together with pathology reports. Finally, a total of 16 patients diagnosed and treated for rectal cancer between November 2015 and January 2019 were curated from a colorectal surgery database at the VAMC. Of these, 11 patients met our inclusion-exclusion criteria of post-nCRT T2w MRIs being available after routine neoadjuvant chemoradiation and prior to surgery, together with pathology reports of excised rectal specimens. Patient enrollment together with inclusion-exclusion criteria is summarized in Figure S3.

### 4.3. Neoadjuvant Treatment and Histopathologic Assessment

All patients included had undergone long-course chemoradiation therapy prior to the restaging MR imaging exam. Radiation therapy had involved 45 to 50.4 Gy in 25 to 28 fractions over 5 to 6 weeks, with concomitant chemotherapy consisting of oral Capecitabine 825 to 850 mg/m^2^ (BID) on days of radiation therapy. Dosages and durations varied slightly at each institution, though the regimen was the same. All patients underwent a proctectomy at a median of 28 days (range: 6–83 days) after the end of nCRT.

As part of routine clinical protocol, pathologists at each institution had assessed and recorded tumor-node-metastasis (ypT-N-M) staging of the excised specimens according to AJCC guidelines [46] into clinical reports for each patient; which was curated during retrospective data collection. This pathologic stage assessment of post-surgical specimens was used as the ground-truth reference. As all patients had undergone standard-of-care chemoradiation based on clinical staging, tumor down-staging was defined as ypT0–2, i.e., a lower pathologic stage than the original clinical stage (also implying minimal or dying tumor within the rectal wall). Table 5 summarizes the study population accrued from all three institutions.

### 4.4. Annotation and ROI Identification on Post-nCRT T2w MRI Datasets

**Figure 5:**
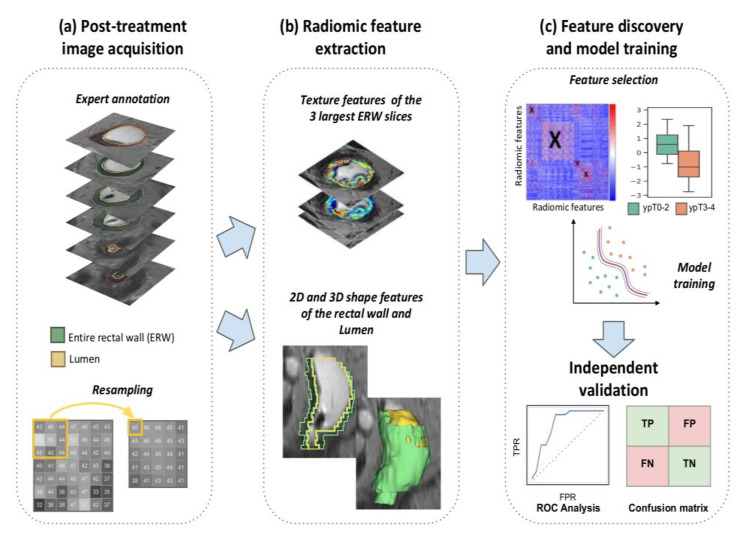
Overview of radiomics pipeline for evaluating pathologic tumor stage regression via post-nCRT T2w MRI.

Based on available clinical, pathologic, and radiology reports (as well as any additional imaging planes and sequences), an expert radiologist at each institution manually annotated two regions of interest (ROI) on each post-nCRT T2w MRI dataset: (i) the entire rectal wall, and (ii) the lumen; via hand-annotation tool in 3D Slicer [47]. They additionally identified the sub-volume of the rectal wall comprising the primary treated tumor region in each dataset. This sub-volume was denoted *R^P^*, the entire rectal wall was denoted *R^E^*, and the lumen was denoted *R^L^*. The discovery cohort was annotated by RP (20 years of body imaging experience) while AP (11 years of experience) and JG (5 years of experience) annotated data from each institution in the validation cohort. To account for differences in voxel resolution across the three institutions (see Table 1), all T2w MRI datasets were linearly resampled to the most consistently occurring resolution in the discovery cohort (0.781 mm × 0.781 mm × 4.0 mm) using 3D Slicer. An overview of the entire radiomics analysis workflow is depicted in Figure 5.

### 4.5. Radiomic Texture and Shape Feature Extraction

Computerized extraction of radiomic texture descriptors was performed using in-house software implemented on MATLAB 2018a (MathWorks, Natick, MA, USA). To ensure that texture descriptors were used to characterize the primary treated tumor region alone, *R^P^* was further limited to a sub-volume comprising the largest annotated 2D section of the primary rectal wall together with two adjacent sections (three consecutive 2D sections total). This was the smallest sub-volume of treated tumor that was consistently available for all patients and thus accounted for varying sizes of *R^P^* among patients. A total of 191 textural descriptors were, thus, computed on a pixel-wise basis within *R^P^* as summarized in Table 6 together with their relevance for quantifying tumor stage regression in rectal cancers. Table S2 additionally provides IBSI (Image Biomarkers Standardization Initiative) [48] compliant definitions for all textural descriptors extracted in this study. First-order statistics (mean, variance, kurtosis, and skewness) were then calculated from each texture feature, resulting in 764 radiomic texture descriptors.

Computerized extraction of radiomic shape descriptors was implemented based on the Insight Segmentation and Registration Toolkit (ITK) (www.itk.org) and MATLAB R2018a (MathWorks). Twenty-five radiomic shape descriptors were extracted for each patient in 3D for both *R^E^* and *R^L^* separately, categorized as (i) contour-based descriptors, which characterize each structure using the object boundary points (e.g., perimeter, elongation, convexity); and (ii) region-based descriptors, which characterize each structure based on the object interior (e.g., area, volume, compactness). Four additional 3D descriptors were computed to quantify the relationship between *R^E^* and *R^L^*, based on taking the difference between diameter-based descriptors. To quantify how shape morphology varied across the entire volume, 2D descriptors were computed for each 2D section and the difference between 2D descriptors from each pair of consecutive sections in the entire volume was computed (done separately for *R^E^* and *R^L^*). First-order statistics (mean, variance, kurtosis, and skewness) were then extracted across all section-based descriptors, yielding a total of 72 2D features. In total, this resulted in a set of 198 shape radiomic descriptors for each patient. Table 7 summarizes all 2D and 3D shape descriptors computed in this study.

### 4.6. Identifying Relevant Radiomic Features Associated with Pathologic Stage after nCRT

Feature normalization was applied to all radiomic features by subtracting the mean and dividing by the mean absolute deviation, resulting in each feature vector having a mean of 0 and mean absolute deviation of 1. This resulted in a normalized radiomic texture feature vector (denoted *F^T^*, extracted from *R^P^*) and a normalized radiomic shape feature vector (denoted *F^S^*, based on concatenating features from both *R^E^* and *R^L^*, respectively).

Radiomic feature selection was implemented via a two-stage process, with methodological choices based on previous large-scale comparisons of feature selection schemes [54,55]. First, a combination of significance testing and correlation testing was implemented [54] to individually prune *F^T^* and *F^S^* in order to remove potentially redundant features (whose correlation coefficient was >0.6 [56]). The resulting pruned feature sets were denoted ***F****^T^* and ***F****^S^* for texture and shape features, respectively. Next, the minimum Redundancy Maximum Relevance (mRMR) algorithm was used to identify the subset of radiomic features from each of ***F****^T^* and ***F****^S^* which best differentiated between down-staged (ypT0–2) and non-regressed tumors (ypT3–4). mRMR seeks to fulfill two criteria at the same time, by selecting features that have the maximal mutual information (MI) but ensuring that the selected features stand for those that have the minimum MI with respect to each other. Finally, ***F****^T^* and ***F****^S^*were concatenated and an optimal combination of texture and shape features (denoted ***F****^T+S^*) were identified from this unified feature vector for differentiating between the 2 pathologic stage groupings via mRMR.

### 4.7. Statistical Analysis

Separate experiments were conducted to evaluate each set of T2w radiomic features: ***F****^T^*, ***F****^S^*, and ***F****^T+S^*, via a quadratic discriminant analysis (QDA) classifier as well as a random forests classifier (RFC). To avoid training bias within the discovery cohort, a randomized 3-fold cross-validation scheme was used in which 2 folds were used for feature selection, and the third fold was used for testing the performance of selected features. This was repeated so that each fold was tested on once, and the entire cross validation process was repeated 50 times. Training model performance for each feature set was quantified in terms of the area under receiver–operator curve (AUC) and the Matthews correlation coefficient [57] of each classifier for differentiating ypT0–2 vs. ypT3–4, averaged across all cross-validation runs. 

The radiomic descriptors within each of ***F****^T^*, ***F****^S^*, and ***F****^T+S^,* were ranked based on how frequently they appeared across all cross-validation runs. Top-ranked features were used to construct a final classifier model (via both QDA and RFC) which was then evaluated in hold-out fashion on the validation cohort. To fully estimate classification performance in the validation cohort, confusion matrices were generated for the best performing classifier model based on each of ***F^T^***, ***F****^S^*, and ***F****^T+S^*; at the optimized threshold determined on the training cohort. Sex differences in model performance [58] in terms of both classifier AUC and MCC were additionally calculated for sex-specific subgroups (male versus female).

The final number of radiomic descriptors selected to build this classifier model when using each of each of ***F****^T^*, ***F****^S^*, and ***F****^T+S^*, was varied from 4 to 6 features in order to prevent overfitting and the “curse of dimensionality”. Robustness of top-ranked radiomic features were also assessed with respect to MR scanner strength by statistically comparing the feature values between all 3.0 T and 1.5 T scans (via Wilcoxon rank sum testing) across both discovery and validation cohorts. Sensitivity of top-ranked radiomic features with respect to expert annotations was evaluated for a subset of 20 patients (10 each from discovery and validation cohorts) for which 2 radiologists (AP and JG) had independently provided annotations of the entire rectal wall and lumen. Note that a subset was utilized due to the fact of time constraints in obtaining a second set of complete expert annotations. Radiomic feature values computed from each expert annotation (for each of *R^E^* and *R^L^*) were compared in pairwise fashion using Wilcoxon ranksum testing (*p* ≤ 0.05 used as the threshold for significant differences) as well as the inter-class correlation coefficient (ICC, values closer to 1 indicate higher correlation). Additionally, overlap between the 2 sets of expert annotations was measured using the dice similarity coefficient (DSC), for both *R^E^* and *R^L^*. 

To further evaluate the performance of combining features within ***F****^T+S^* in comparison to ***F****^T^* and ***F****^S^* individually, each feature set was projected into 3 dimensions via the t-SNE algorithm [59], with random initialization, 30 nearest neighbors and 1000 iterations by Euclidean metric; using the validation cohort. As this tool has been shown to optimally preserve non-linear high-dimensional relationships into lower-dimensional spaces, naturally occurring clusters in the data could be easily visualized via a 3D scatter plot of each *t*-SNE space. Quantitative evaluation of these clusters was done via consensus clustering of the three different *t*-SNE projections using the ConsensusClusterPlus package in R [60,61], with 1000 iterations of hierarchical consensus clustering (*k* = 2) by Pearson distance and 80% random patient resampling between runs. Clustering results were visualized in a consensus cluster heatmap where the blue shading indicated the frequency with which a pair of patients was clustered together across all runs. Clustering results were also compared against ypT groupings to quantify the ability of top features to identify each of the two groups in an unsupervised fashion, within the validation cohort. 

## 5. Conclusions

Restaging rectal cancer after neoadjuvant therapy is currently one of the most significant clinical challenges, since it provides the possibility of changing the planned surgical treatment based on accurately determining tumor regression after nCRT. In this work, we presented the first multi- institutional study for identifying radiomic texture and shape features from routine post-nCRT T2w MRI that were found to be associated with rectal cancer patients who achieved pathologic tumor down-staging after chemoradiation. The most relevant features identified were quantitative measurements of specific heterogeneity patterns and structural distensions of the rectal wall, and the resulting radiomic model maintained its performance across data from three different institutions as well as across different magnetic field strengths. This set of radiomic texture and shape descriptors appear to be driven by intuitive histopathological and physiological differences between pathologic stage groupings of rectal tumors after nCRT. Future work will include integrating our analysis with pre-treatment imaging prediction models [62] for a more comprehensive assessment of tumor evolution after chemoradiation in rectal cancers. We also plan to evaluate the performance of our predictor in a more prospective setting, as well as across different platforms and implementations to confirm generalizability of identified radiomic descriptors. These findings potentially hold significant clinical application as they could be used as a non-invasive tool for post-treatment identification of rectal cancer patients who could benefit from minimally invasive surgical management, based on more accurate evaluation of pathologic tumor response after chemoradiation.

## Figures and Tables

**Table 1:** Summary of imaging parameters for post-nCRT T2w MRI scans used in this study.

Imaging Parameter	Institution 1 UHCMC (*n* = 52)	Institution 2 CCF (*n* = 31)	Institution 3 VAMC (*n* = 11)
In-plane Resolution (mm)	0.256–0.977	0.313–0.898	0.398–0.938
Slice Thickness (mm)	3.0–5.0	3.0–6.0	3.0–8.0
Field of view (px)	224–576 × 224–576 × 20–57	256–640 × 252–640 × 13–79	234–576 × 256–528 × 24–50
Repetition Time (ms)	3253–12690	3400–13333	3420–7200
Echo Time (ms)	67–110	84–166	80–100
Sequence	TSE	TSE	FSE
*Magnet Strength*			
3 T	51	3	8
1.5 T	1	28	3
*Scanner*			
Siemens Symphony		6	
Siemens Avanto		14	
Siemens Espree		3	
Siemens Aera		4	
Siemens Skyra		3	
Siemens Verio	39		
Philips Achieva		1	8
Philips Medical System Ingenuity	5		
Philips Healthcare Ingenia	8		
Toshiba Titan			2
Unknown			1
*Imaging Plane Axial Through Tumor*			
Transverse	43	28	10
Coronal	9	3	1
*Gel use*	Yes	Yes	No

UHCMC = University Hospitals Cleveland Medical Center; CCF = Cleveland Clinic Foundation, VAMC = Louis Stokes Veterans Affairs Medical Center.

**Table 2:** Top-ranked radiomic descriptors within each of ***F****^T^*, ***F****^S^*, and ***F****^T+S^*, together with their *p*-value from Wilcoxon ranksum testing (unadjusted) between ypT0–2 from ypT3–4 patient groupings. Features are ranked based on selection frequency across 50 runs of 3 fold cross-validation. Note that ***F****^T+S^* comprises a combination of top-ranked descriptors from each of ***F****^T^*and ***F****^S^*.

Rank	*F^T^*	*F^S^*	*F^T+S^*
1	Median Gradient Sobel xy (*p* = 0.0002)	3D Compactness Entire Rectal Wall (*p* = 0.003)	Median Gradient Sobel xy (*p* = 0.0002)
2	Skewness Gradient dy (*p* = 0.0007)	Skewness - 2D Eccentricity Lumen (*p* = 0.004)	3D Compactness Entire Rectal Wall (*p* = 0.003)
3	Median Laws L3S3 (*p* = 0.0009)	Variance - 2D Convexity Entire Rectal Wall (*p* = 0.0009)	Variance - 2D Convexity Entire Rectal Wall (*p* = 0.0009)
4	Median CoLlAGe sum-av ws = 5 (*p* = 0.002)	Mean - 2D Compactness Entire Rectal Wall (*p* = 0.002)	Median Laws L3S3 (*p* = 0.0009)
5	Median Haralick sum-av ws = 3 (*p* = 0.006)	Variance - 2D Minor Axis Length Lumen (*p* = 0.0009)	Skewness Gradient dy (*p* = 0.0007)
6	Variance Haralick sum-av ws = 3 (*p* = 0.006)	Kurtosis - 2D Major Axis Length Lumen (*p* = 0.02)	Median CoLlAGe sum-av ws = 5 (*p* = 0.002)

***F**^T^* = Normalized radiomic texture feature vector;***F**^S^* = normalized radiomic shape feature vector; ***F**^T+S^* = combination of texture and shape feature vector.

**Table 3:** Statistical comparison of top-ranked texture and shape radiomic descriptors between different magnetic field strengths. *p*-values computed via Wilcoxon rank sum testing.

Ranked Feature	1.5 T Median (IQR)	3 T Median (IQR)	Unadjusted *p*-Value
Median Gradient Sobel xy	0.64 (−0.05–1.40)	0.31 (−1.34–1.12)	0.127
3D Compactness: ERW	−0.43 (−2.09–1.77)	−0.64 (−1.42–0.17)	0.921
Variance—2D Convexity ERW	−2.26 (−2.72–−0.91)	−1.37 (−2.31–0.34)	0.015
Median Laws L3S3	0.79 (−0.80–1.57)	0.60 (−1.04–1.65)	0.814
Skewness Gradient dy	0.26 (−0.77–0.96)	0.30 (-1.12–0.88)	0.423
Median CoLIAGe sum-av ws = 5	−0.21 (−1.07–0.84)	−0.50 (−1.31–1.03)	0.789
Median Haralick sum-av ws = 3	−0.97 (−1.82–0.29)	−0.03 (−0.9–1.21)	0.014
Variance Haralick sum-av ws = 3	0.05 (−0.95–0.79)	−0.54 (−1.34–0.71)	0.369
Skewness—2D Eccentricity Lumen	0.11 (−1.54–1.04)	0.06 (-0.67–0.99)	0.510
Mean—2D Compactness ERW	−0.2 (−0.98–0.58)	0.37 (−0.97–1.17)	0.166
Variance—2D Minor Axis Length Lumen	−0.03 (−0.80–1.82)	−0.45 (−1.49–0.43)	0.030
Kurtosis—2D Major Axis Length Lumen	−1.33 (−1.81–0.09)	−0.60 (−1.64–1.05)	0.423

**Table 4:** Statistical comparison of top-ranked texture and shape radiomic descriptors between 2 independent expert annotations on a subset of 20 patients (from across discovery and validation cohorts). *p*-values computed via Wilcoxon rank sum testing. ICC: Intra-class Correlation Coefficient.

Radiomic Feature	Unadjusted *p*-Value	ICC
Median Gradient Sobel xy	0.457	0.549
3D Compactness: ERW	0.776	0.923
Variance—2D Convexity ERW	0.441	0.375
Median Laws L3S3	0.693	0.908
Skewness Gradient dy	0.962	0.484
Median CoLIAGe sum-av ws = 5	0.602	0.829
Median Haralick sum-av ws = 3	0.912	0.947
Variance Haralick sum-av ws = 3	0.079	0.745
Skewness - 2D Eccentricity Lumen	0.925	0.473
Mean—2D Compactness ERW	0.903	0.842
Variance—2D Minor Axis Length Lumen	0.903	0.831
Kurtosis—2D Major Axis Length Lumen	0.285	0.695

**Table 5:** Summary of demographic and pathologic information from multi-institution data cohort used in this study.

Clinical Variable	Inst. 1 UHCMC (*n* = 52)	Inst. 2 CCF (*n* = 31)	Inst. 3 VAMC (*n* = 11)
*Gender*			
Male	30	20	11
Female	22	11	0
Age at diagnosis (yrs)	62.8 ± 13.6	58.2 ± 11.4	65.8 ± 12.0
Rectal wall volume (cm^3^)	43.1 ± 33.6	62.4 ± 66.1	35.9 ± 17.6
Lumen wall volume (cm^3^)	40.1 ± 31.1	69.5 ± 43.4	21.8 ± 8.8
**Pathologic Staging**			
*ypN0M0*			
ypT0–2	18	7	5
ypT3–4	15	9	2
*ypN+ or ypM+*			
ypT0–2	4	2	2
ypT3–4	15	13	2

**Table 6:** Texture descriptor families utilized in this study together with physiologic rationale and implementation.

Feature Group	Quantity	Description & Rationale
Histogram measures	21	First-order statistics of the original image signal intensity within local pixel neighborhoods, capturing basic variations in signal intensities due to intermixed tissue types (fibrosis, ulceration, mucosa) after nCRT
Gradient operators [49]	10	Identification of leading gradients and edges in the local signal within small neighborhoods of pixels, likely occurring due to impact of nCRT within the rectal wall
Haralick measures [50]	65	Quantify heterogeneity and entropy of local intensity texture as represented by the gray-level co-occurrence matrix pixel neighborhoods, widely shown to be related to underlying tissue heterogeneity as a result of intermixed treatment effects, residual disease, and irradiated tissue
Gabor operators [51]	35	Responses to Gabor wavelets which are defined at specific unit-length scales (λ = 0.765, 0.128, 1.786, 2.296, and 2.806; corresponding to window sizes 3, 5, 7, 9 or 11 pixels) and orientations (θ = $\frac{\pi }{8},\frac{\pi }{4},\frac{3\pi }{8},\frac{\pi }{2},\frac{5\pi }{8},\frac{3\pi }{4}$), thus capturing multi-scale and multi-oriented variations within the rectal wall
Laws operators [52]	34	Responses to local filters targeting combinations of specific textural patterns in the x- and y-directions. Descriptors include all combinations of 1D filters: level (L), edge (E), spot (S), wave (W), and ripple (R), which have been related to underlying abnormal structures or enhancement patterns
CoLlAGe [53]	26	Captures and exploits local anisotropic differences in voxel-level gradient orientations by assigning every image voxel an entropy value associated with the co-occurrence matrix of gradient orientations, which have been related to reflecting subtle local differences in tissue microarchitecture

nCRT = neoadjuvant chemoradiation therapy; CoLlAGe = co-occurrence of local anisotropic gradient orientations.

**Table 7:** Description of 2D and 3D shape radiomic descriptors extracted and utilized in this study.

Feature Name	Description	2D	3D
Contour-Based			
Axis length	Length of a line drawn through the center of an ellipse (2D) or sphere (3D) that has the same normalized second central moments as the object	x	x
Convexity	Ratio between the convex perimeter and the perimeter of the original object	x	
Convex perimeter	Length of the outline of the convex object (smallest convex polygon that can contain the object)	x	
Eccentricity	Ratio of the distance between the foci of the ellipse (2D) or sphere (3D) and its major axis length, measuring how much a conic section deviates from being circular	x	x
Elongation	Ratio between the minor and the major axis, measuring the aspect ratio of the object	x	x
Equivalent diameter	Diameter of a circle that has the same area as the object	x	
Equivalent ellipsoid diameter	Diameter of an ellipse that has the same second-moments as the object		x
Equivalent spherical radius	Radius of a sphere that has the same second-moments as the object		x
Equivalent spherical perimeter	Perimeter of a sphere that has the same second-moments as the object		x
Flatness	Measure that describe if the surface of the object is flat or if it has raised areas or indentations		x
Orientation	Angle between the x-axis and the major axis of the ellipse (2D) or sphere (3D) that has the same second-moments as the object	x	x
Perimeter	Length of the outline of the object	x	
**Region-based**			
Area	Measure of the number of pixels in a 2D object	x	
Area of bounding box	Measure of the number of pixels in the bounding box (smallest rectangle containing the region)	x	
Compactness	Ratio between the area (2D) of the object and the area of a circle with the same perimeter	x	x
Convex area	Measure of the number of pixel in the convex hull (the smallest convex polygon that can contain the region)	x	
Elongation of the bounding box	Ratio between the minor and the major axis of the bounding box (smallest rectangle containing the region), measuring the aspect ratio of the object	x	
Elongation shape factor	Square root of the ratio of the two second moments of the object around its principal axes		x
Extent	Ratio between pixels in the original object and pixels in the bounding box (smallest rectangle containing the region)	x	
Filled area	Number of pixels in the filled object (original object with all the holes filled)	x	
Principal moments	Measures that describe the moments of inertia at center of mass		x
Roundness	Ratio between the area (2D) or volume (3D) of the object and the area of a circle (2D) or sphere (3D) with the same convex perimeter	x	x
Solidity	Density of the object measured as proportion of the pixels in the convex object (smallest convex polygon that can contain the object) that are also in the original object	x	
Volume	Measure of the number of pixels in a 3D object		x

## Supplementary Materials

The following are available online at https://www.mdpi.com/2072-6694/12/8/2027/s1, Figure S1: Random forest model AUC performance while varying the number of radiomic features used (*x*-axis) when evaluated on (a) discovery, and (b) validation cohorts. The different colors and symbols correspond to ***F****^T^* (orange),***F**^S^* (blue), and ***F****^T+S^* (green); respectively. Error bars on (a) reflect ± 1 standard deviation of AUC in cross-validation on the discovery cohort, Figure S2: Box plots of (a)–(f) top 6 radiomics descriptors in ***F****^T+S^*; when comparing ypT0-2 (green) to ypT3-4 (orange) tumors for the three different institutions involved in this study. Also shown are confusion matrices for the validation cohort comprising (g) Inst. 2 (CCF), and (h) Inst. 3 (VAMC) at the optimized threshold, Figure S3: CONSORT style flow diagram of patient enrollment, eligibility, and exclusion criteria of the multi-institutional dataset used in this study, Table S1: QDA model performance for ***F****^T+S^* in sex-specific subgroups within discovery and validation cohorts, Table S2: Implementation details of radiomic texture features utilized in this study.
