# Supplementary material for: Radiomic Texture and Shape Descriptors of the Rectal Environment on Post-Chemoradiation T2-Weighted MRI are Associated with Pathologic Tumor Stage Regression in Rectal Cancers: A Retrospective, Multi-Institution Study
Source: Cancers (Basel). 2020 Jul 24;12(8):2027. doi: 10.3390/cancers12082027 (PMC7463898; doi:10.3390/cancers12082027)
Supplement: Supplementary file 1 [file cancers-12-02027-s001.pdf]

Supplementary Materials

Radiomic Texture and Shape Descriptors of the Rectal Environment on Post-Chemoradiation T2-Weighted MRI are Associated with Pathologic Tumor Stage Regression in Rectal Cancers: A Retrospective, Multi-Institution Study

Charlems Alvarez-Jimenez, Jacob T. Antunes, Nitya Talasila, Kaustav Bera, Justin T. Brady, Jayakrishna Gollamudi, Eric Marderstein, Matthew F. Kalady, Andrei Purysko, Joseph E. Willis, Sharon Stein, Kenneth Friedman, Rajmohan Paspulati, Conor P. Delaney, Eduardo Romero, Anant Madabhushi and Satish E. Viswanath

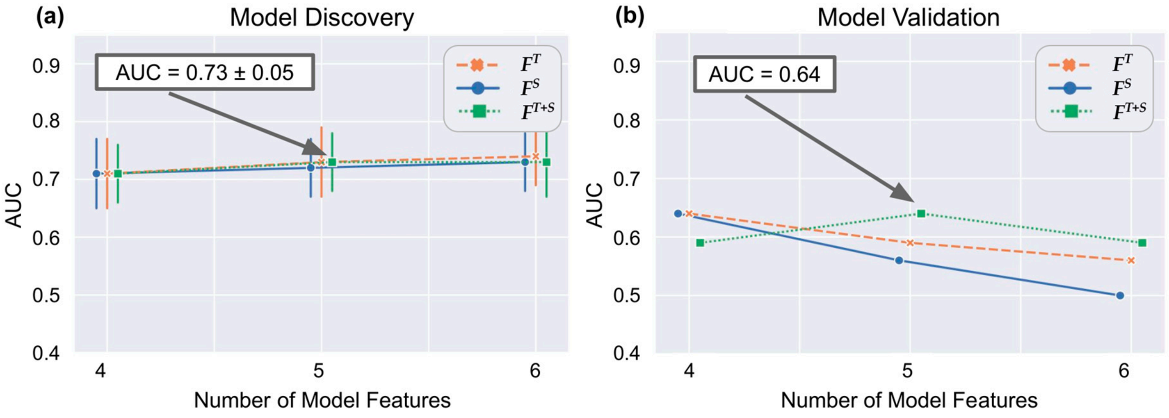

Figure S1. Random forest model AUC performance while varying the number of radiomic features used (X-axis) when evaluated on (**a**) discovery, and (**b**) validation cohorts. The different colors and symbols correspond to $\boldsymbol{F}^{T}$ (orange), $\boldsymbol{F}^{S}$ (blue), and $\boldsymbol{F}^{T+S}$ (green); respectively. Error bars on (a) reflect ± 1 standard deviation of AUC in cross-validation on the discovery cohort.

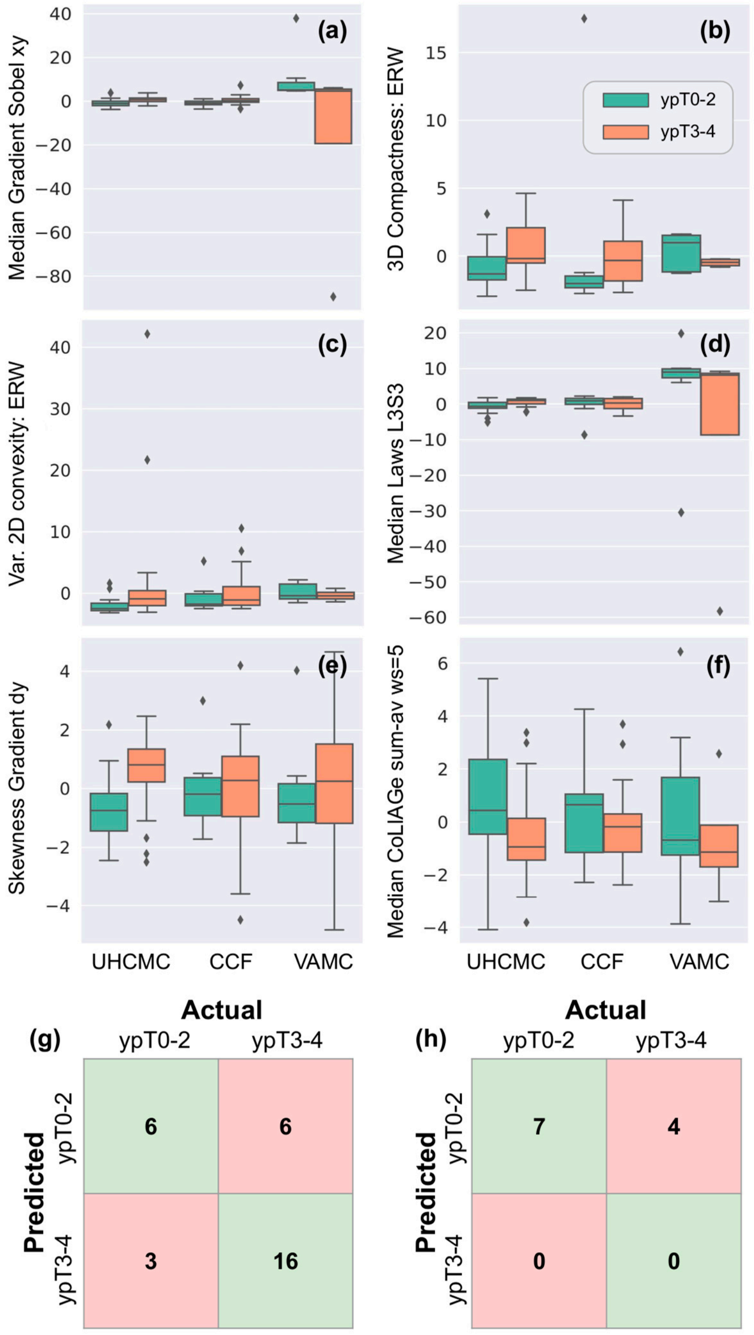

Figure S2. Box plots of (**a**–**f**) top 6 radiomics descriptors in F^{T+S}; when comparing ypT0-2 (green) to ypT3-4 (orange) tumors for the 3 different institutions involved in this study. Also shown are confusion matrices for the validation cohort comprising (**g**) Inst. 2 (CCF), and (**h**) Inst. 3 (VAMC) at the optimized threshold.

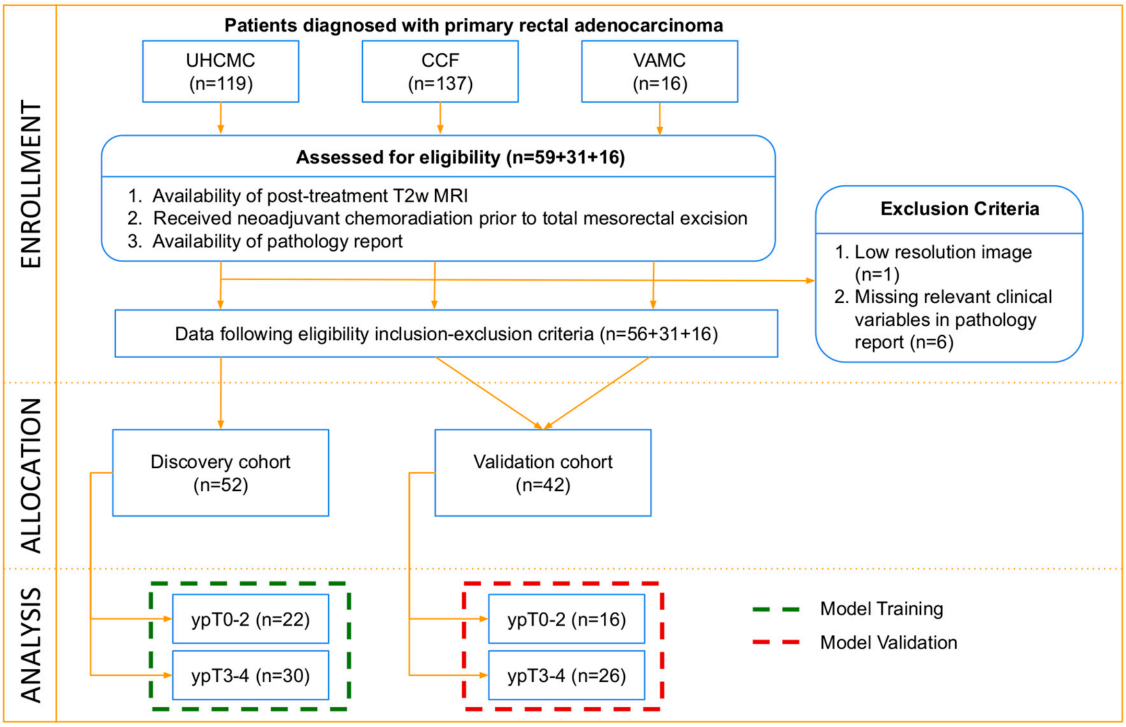

Figure S3. CONSORT style flow diagram of patient enrollment, eligibility, and exclusion criteria of the multi-institutional dataset used in this study.

Table S1. QDA model performance for F^{T+S} in sex-specific subgroups within discovery and validation cohorts.

	Discovery			Validation		
	**Both**	**Male**	**Female**	**Both**	**Male**	**Female**
Patients	52	30	22	42	31	11
AUC	0.67 ± 0.06	0.67 ± 0.08	0.68 ± 0.09	0.73	0.74	0.67
MCC	0.36 ± 0.1	0.36 ± 0.15	0.36 ± 0.18	0.42	0.51	0.15

Table S2. Implementation details of radiomic texture features utilized in this study.

Feature Category	Implementation Details
Intensity Histogram	1) 1 distribution of global intensities in region of interest<br>2) 4 distributions calculated from m x m window size of kernels; m ϵ {3 5, 7, 9, 11}<br>i. Mean of m x m kernel<br>ii. Median of m x m kernel<br>iii. Range of m x m kernel<br>iv. Standard deviation of m x m kernel
Laws	1) m x m window size of kernels; m ϵ {3 5}<br>2) the 'same' option in MATLAB command **conv** was used to trim the outer part of the convolution and return only the central part, which was the same size as the input<br>3a) 1D Kernels used (for window size 3):<br>L (Level) = [1 2 1]<br>E (Edge) = [-1 0 1]<br>S (Spot) = [-1 2 -1]<br>3b) 1D Kernels used (for window size 5):<br>L (Level) = [1 4 6 4 1]<br>E (Edge) = [-1 -2 0 2 1]<br>S (Spot) = [-1 0 2 0 -1]<br>W (Wave) = [-1 2 0 -2 1]<br>R (Ripple) = [1 -4 6 -4 1]<br>4) Two 1-D kernels were combined via matrix multiple to generate either a 3 x 3 kernel (for window size 3) or 5 x 5 kernel (for window size 5) all permutations of 1-D kernels were implemented
Gabor	The real component of the Gabor filter response in 2D at a particular (x, y) location was defined as:<br>$g_{\lambda,\theta\varphi,\sigma,\gamma}(x,y) = exp\left(-\frac{x'^2+\gamma^2 y'^2}{2\sigma^2}\right) cos\left(2\pi\frac{x'}{\lambda}+\varphi\right)$<br>where<br>$x' = xcos\theta_{xy} - ysin\theta_{xy}$<br>$y' = xsin\theta_{xy} + ycos\theta_{xy}$<br>$\sigma = \frac{\lambda(2^B+1)}{\pi(2^B-1)}\sqrt{\frac{ln(2)}{2}}$<br>The necessary parameters are defined below along with their implemented values:<br>• θ_{xy}: orientation in x-y plane; θ_{xy} ϵ $\{0, \frac{\pi}{6}, \frac{\pi}{3}, \frac{\pi}{2}, \frac{2\pi}{3}, \frac{5\pi}{6}\}$ radians<br>• γ: $anisotropic\ scaling\ factor\ in\ y\ (for\ isotropy, fixed\ at\ \gamma = 1)$<br>• B: bandwidth, or half-response spatial frequency; fixed at $B = 1$ (therefore $\sigma \approx 0.56\lambda$)<br>• λ: wavelength of cosine factor; determined such that 7σ would approximate half the window size of a m x m kernel,; λ ϵ {0.3827, 0.6378, 0.8926, 1.1480 1.4142} such that m ϵ {3 5, 7, 9, 11}<br>• σ: specified based on B, λ. Isotropic filter, so σ same in all directions<br>• φ: phase offset; fixed at $\varphi = 0$ in all directions
Haralick	1) image quantization approach - Uniform (i.e. equal distances between original gray levels and quantized bins)<br>2) number of bins - 128 (i.e. 128 gray levels)<br>3) offset - 1 (i.e., search D = 1 pixels away from pixel of interest)<br>4) number of directions - 4 directions (bi-directional),<br>- right diagonal: 45 or 135degrees<br>- vertical: 90 or 270degrees<br>- left diagonal:135 or 315degrees<br>- horizontal: 0 or 180degrees<br>5) extraction method - symmetrically<br>6) aggregation approach for final feature estimation<br>- For each pixel of interest, gray-level co-occurrence (GLCM) calculations were summed among all pixels within a fixed m x m x m window centered around the pixel, to create a single co-occurrence matrix. Varying window sizes were tested (m ϵ {3, 5, 7, 9, 11} pixels).<br>- Features were extracted from the co-occurrence matrix for each pixel of interest, yielding 13 GLCM feature representations for each pixel of interest (visualized as heatmaps)
Gradient	10 kernels of size 3 x 3 designed to capture unique directional gradients<br>- Gradient sobel x: $[-1\ 0\ 1\ -2\ 0\ 2\ -1\ 0\ 1\ ]$<br>- Gradient sobel y: $[1\ 2\ 1\ 0\ 0\ 0\ -1\ -2\ -1\ ]$

	- Gradient sobel xy : $[0\ 1\ 2\ -1\ 0\ 1\ -2\ -1\ 0\,]$<br>- Gradient sobel yx: $[2\ 1\ 0\ 1\ 0\ -1\ 0\ -1\ -2\,]$<br>- Gradient x: $\frac{dF}{dx}$ (F is image intensities); MATLAB command **gradient**<br>- Gradient y: $\frac{dF}{dy}$; MATLAB command **gradient**<br>- Gradient magnitude: $\sqrt{\frac{dF}{dx}^2 + \frac{dF}{dx}^2}$<br>- Gradient dx: MATLAB command **conv2**(img, [-1 1],'same')<br>- Gradient dy: MATLAB command **conv2**(img, [-1; 1],'same')<br>- Gradient diagonal: MATLAB command **conv2**(img, [-1 0; 0 1], 'same')
CoLlAGe	1) MATLAB command **gradient** applied to image to get gradients between each pair of neighbouring pixels in horizontal, vertical and diagonal directions (bi-directional)<br>2) The dominant orientation (based on maximum gradient magnitude) is identified and assigned to the pixel in radians on the scale [0, 2π), generating a pixel-wise map of gradient orientations<br>3) GLCM computations (from Haralick features) are calculated off the gradient orientation maps, using the same methodology aforementioned, in either 3 x 3 or 5 x 5 windows<br>4) Features were extracted from the co-occurrence matrix for each pixel of interest, yielding 13 GLCM feature representations for each pixel of interest (visualized as heatmaps) at each window size

© 2020 by the authors. Licensee MDPI, Basel, Switzerland. This article is an open access article distributed under the terms and conditions of the Creative Commons Attribution (CC BY) license (http://creativecommons.org/licenses/by/4.0/).
